# Editorial: Monitoring endogenous GPCRs: lessons for drug design

**DOI:** 10.3389/fphar.2015.00146

**Published:** 2015-07-14

**Authors:** Dominique Massotte

**Affiliations:** Institut des Neurosciences Cellulaires et IntégrativesStrasbourg, France

**Keywords:** G protein-coupled receptors, biased signaling, heteromers, chronic pain, mood disorders

G protein-coupled receptors (GPCRs) are integral membrane proteins forming the fourth largest superfamily in the human genome. Many of these receptors play key physiological roles and several pathologies have been associated with receptor functional abnormalities. GPCRs therefore represent important goals for drug design in pharmaceutical companies since they constitute the target of about one third of the drugs currently on the market. However, endogenous GPCRs are most often difficult to study because of a lack of tools to target them specifically and single out their response to physiological or drug-elicited stimulations. To date, studies mostly focused on recombinant receptors expressed in a variety of cellular models that do not always closely reflect the receptor natural environment and often deal with levels of expression exceeding by far physiological ranges. Recent technological developments have improved our ability to visualize endogenous GPCRs and to address their signaling properties. Data suggest that the receptor may embrace a different fate depending on the ligand. This so-called biased signaling is getting growing importance in the GPCR field. Similarly, increasing attention is given to the concept of heteromerization that corresponds to the physical association of two receptor types resulting in new signaling properties. Investigating endogenous receptor activation and subsequent intracellular redistribution or addressing changes induced by drug-elicited stimulation from molecular and cellular events to integrated response is thus crucial for the development of new pharmacological tools and strategies. In this topic, timely overview as well as original reports present new tools, including genetically modified animals, and techniques available to track expression and signaling of endogenous GPCRs.

Brogi et al. (2014) review novel approaches in medicinal chemistry for class A GPCRs that all aim at more efficacy with less side effects. They are ranging from *in silico* studies for increased ligand selectivity and affinity to new orientations in ligand development including biased agonists that favor specific signaling cascades such as G protein or beta-arrestin dependent pathways, allosteric modulators or bivalent ligands that target heteromers. Thompson et al. (2014) illustrate the concept of biased agonism at the level of the endogenous somatostatin and opioid systems in the gut. In the case of opioid receptors, biaised agonism could be achieved through heteromer formation. In this context, Gonzalez-Maeso (2014) provides a brief overview of the techniques currently available to establish physical proximity between receptors *in vivo*, which represents the first criterion to postulate heteromer formation. In the same line, Gomes et al. (2014) introduce the generation of heteromer selective antibodies by substractive immunization strategy and discuss their use to get insight into class A heteromer-specific signaling *in vivo*. Moving to genetically modified animals, Ceredig and Massotte (2014) review the contribution of knock-in mice that express fluorescent proteins to neuroanatomy. The authors highlight the role of knock-in animals expressing fluorescent receptors for linking receptor trafficking, desensitization and behavioral output and for mapping receptor neuronal co-expression as a first hint toward *in vivo* heteromers. Knock-out animals on the opposite are deficient for a given receptor but proved powerful to decipher the specific role of a given GPCR in various physiopathological conditions. This is exemplified by Befort (2015) who reviews the relative contribution of opioid and cannabinoid receptors and their interactions in the context of reinforcing behaviors and discusses the limitations of the approach. Genetically modified animals are also powerful tools to address GPCR signaling. As an example, GCaMP transgenic mice express engineered proteins containing Ca^2+^ binding motifs within a circularly permutated variant of the green fluorescent protein that undergo a conformational change upon elevation of intracellular Ca^2+^. Partridge (2015) reviews the use of this Ca^2+^ sensor to monitor *in vivo* activation of Gq/11 coupled GPCRs in response to pharmacological stimulation. Alternatively, Bagley (2014) reports an original study that illustrates the utility of classical approaches such as electrophysiology as another powerful tool to identify the specific impact of a given receptor on neuronal activity. She addresses the identity of the Gi/o coupled receptor responsible for protein kinase A (PKA)-dependent increase of the GABA transporter GAT-1 in the periaqueductal gray, a phenomenon underlying increased GABAergic neuronal excitability and synaptic GABA release during opiate withdrawal. Combining perforated patch recording with selective pharmacological stimulation, Bagley clearly demonstrates that PKA dependent increase in GAT 1 is promoted by opioid receptor activation and not GABA_B_ receptors possibly due to differential subcellular distribution of the two receptors within the neuron. Chen et al. (2014) also report a novel approach to monitor PKA activity in brain tissue by fluorescence lifetime imaging microscopy (FLIM) using two-photon microscopy using their newly developed PKA sensor FLIM-AKAR. FLIM-AKAR can be transfected or virally encoded for *in vivo* expression. The latter can be controlled by cre-dependent elements to target specific neuronal populations. This sensor reports the balance of PKA and phosphatase activity with less pH sensitivity and a broader dynamic range. Moreover, FLIM-AKAR being highly diffusible enables monitoring of PKA activity in dendritic spines. Finally, two reviews broach the functional role of endogenous opioid receptors. Cahill et al. (2014) expand our knowledge of the role of the kappa opioid receptor and its endogenous ligand dynorphin. The authors review evidence of the implication of the kappa-dynorphin system in the negative aspects related to pain, highlighting possible contribution in the high comorbidity of mood disorders associated with chronic neuropathic pain. Allouche et al. (2014) review the various mechanisms by which opioid receptors desensitize including aspects related to biased agonism and discuss their impact on the development of opiate tolerance.

Altogether, the topic covers various conceptual and technical approaches at the molecular, cellular or integrated level that can be generalized to challenge the functional role of endogenous class A GPCRs and to gather critical insight for novel therapeutic strategies.

## Conflict of interest statement

The author declares that the research was conducted in the absence of any commercial or financial relationships that could be construed as a potential conflict of interest.
